# First molecular detection and phylogenetic characterization of *Balantioides coli* in lowland tapir (*Tapirus terrestris*)

**DOI:** 10.1007/s00436-026-08707-0

**Published:** 2026-06-11

**Authors:** Breno Torres da Silva, Camila Souza Carvalho Class, Rodrigo Hidalgo Friciello Teixeira, Maron Galliez, Elan Cardozo Paes de Almeida, Laís Lisboa Corrêa, Laís Verdan Dib, Maria Regina Reis Amendoeira, Alynne da Silva Barbosa

**Affiliations:** 1 Parasitology Laboratories, Biomedical Institute, Fluminense Federal University, Outeiro São João Batista s/n, Niterói, Rio de Janeiro 24020-141 Brazil; 2 Municipal Zoological Park “Quinzinho de Barros” (PZMQB), University of Sorocaba (UNISO), Teodoro Kaisel Street, 883, Sorocaba, São Paulo Brazil; 3 Graduate Program in Wild Animals, São Paulo State University “Júlio de Mesquita Filho” (UNESP – Botucatu), Botucatu, São Paulo Brazil; 4 Federal Institute of Rio de Janeiro (IFRJ), Senador Furtado Street, 121, Maracanã, Rio de Janeiro, Rio de Janeiro 20270021 Brazil; 5 Multidisciplinary Biomedical Research Laboratory, Nova Friburgo Health Institute, Dr. Silvio Henrique Braune Street, No. 22, Nova Friburgo, Rio de Janeiro 28625-650 Brazil; 6 Protozoology Laboratory, Oswaldo Cruz Institute, Fiocruz, Carlos Chagas Pavilion, 2nd Floor, Room 208D, Brasil Avenue, 4365, Rio de Janeiro, 21045-900 Brazil; 7 School of Health and Wellness Sciences, Souza Marques University Center, Ernani Cardoso Avenue, 335, Rio de Janeiro, 21310-310 Brazil

**Keywords:** *Balantioides coli*, *Tapirus terrestris*, Ciliophora, Wildlife parasites, One Health

## Abstract

As a vulnerable species to extinction, *Tapirus terrestris* has scarce data on gastrointestinal parasites, particularly regarding the occurrence of zoonotic ciliates such as *Balantioides coli*. Therefore, gastrointestinal parasites were investigated in this host using parasitological, in vitro culture, and molecular approaches. A total of 45 fecal samples were analyzed from animals under human care and from free-ranging or reintroduced individuals in southeastern Brazil. Overall parasite positivity reached 68.9%, varying among sampling sites. Protozoa from the phylum Ciliophora represented the most frequent group, occurring in 60% of samples (*p* ≤ 0.05). Nematode forms were identified in 31.1% of samples, predominantly as strongylid-type eggs, as well as eggs consistent with *Trichuris* spp. (4.4%) and members of the family Spiruridae (4.4%). Coccidian oocysts were detected in 20% of samples and were morphometrically compatible with the genus *Eimeria*. In vitro culture enabled the successful isolation of 20 ciliate strains, and PCR amplification targeting the ITS1–5.8SrDNA–ITS2 region confirmed *B. coli* in 24 samples, including isolates obtained directly from feces and in vitro cultures, corresponding to an infection rate of 53.3%. Sequence identity ranged from 98.08% to 100% compared to GenBank references. Phylogenetic analysis grouped all sequences within the *B. coli* clade and identified genetic variants A0 (50%), B0 (37.5%), and B1 (8.3%). Variant A0 predominated and was detected in both *ex situ* and *in situ* populations, whereas B0 and B1 showed more restricted distributions. Additionally, amoebae of the genus *Entamoeba* were detected in 17.8% of samples. These findings represent the first molecular confirmation of *B. coli* infection in *T. terrestris* and reinforce the epidemiological relevance of this host in the circulation of zoonotic protozoa across different ecological contexts.

**Supplementary Information:**

The online version contains supplementary material available at 10.1007/s00436-026-08707-0.

## Introduction

*Tapirus terrestris* is the largest terrestrial mammal in Brazil and plays an important ecological role in tropical ecosystems, particularly through seed dispersal processes that contribute to forest regeneration. However, due to its low reproductive rate, historical anthropogenic pressures, and habitat fragmentation, the species is currently classified as vulnerable to extinction in Brazil (Médici et al. 2012; Ferreguetti et al. 2017). In the state of Rio de Janeiro, local populations remained regionally extinct for approximately a century, with the last historical record reported in 1914. Reintroduction programs initiated in 2018 in the Guapiaçu Ecological Reserve (Cachoeiras de Macacu, Rio de Janeiro) have created new opportunities for ecological and health monitoring of reestablished populations (Galliez et al. 2024).

The global decline of wild mammal populations has been associated with multiple anthropogenic factors, including habitat loss, agricultural expansion, road mortality, environmental pollution, and increasing contact between domestic animals and wildlife (Aranda et al. 2013). In this context, parasitic infections represent an additional factor potentially affecting wildlife health, particularly gastrointestinal parasites that may be introduced or amplified in natural ecosystems through human-mediated environmental changes (Mangini et al. 2002).

Among the parasites reported in tapirs, ciliate protozoa morphologically compatible with *Balantioides coli* have been described in fecal samples. This protozoan is considered a zoonotic parasite capable of infecting several hosts, including pigs, non-human primates, and humans (Barbosa et al. 2018). In humans, infection may range from asymptomatic carriage to severe dysenteric disease (Solaymani-Mohammadi and Petri 2006). Despite previous reports of ciliates in tapirs based on microscopic observations, molecular confirmation of *B. coli* in this host has not yet been demonstrated. Furthermore, morphological identification alone may be unreliable due to similarities with other ciliates of the phylum Ciliophora, such as *Buxtonella sulcata* (Barbosa et al. 2018).

In Brazil, ciliates morphologically compatible with Ciliophora have been previously detected in fecal samples from tapirs kept under human care in zoological institutions (Barbosa et al. 2020; Batista et al. 2021), but their molecular characterization has not yet been performed. Therefore, this study aimed to investigate the frequency and diversity of gastrointestinal parasites in *T. terrestris* from both free-ranging and *ex situ* populations, integrating conventional parasitological, morphological, and molecular approaches, with emphasis on the taxonomic and molecular characterization of ciliates.

## Materials and methods

### Study area

**Fig. 1:**
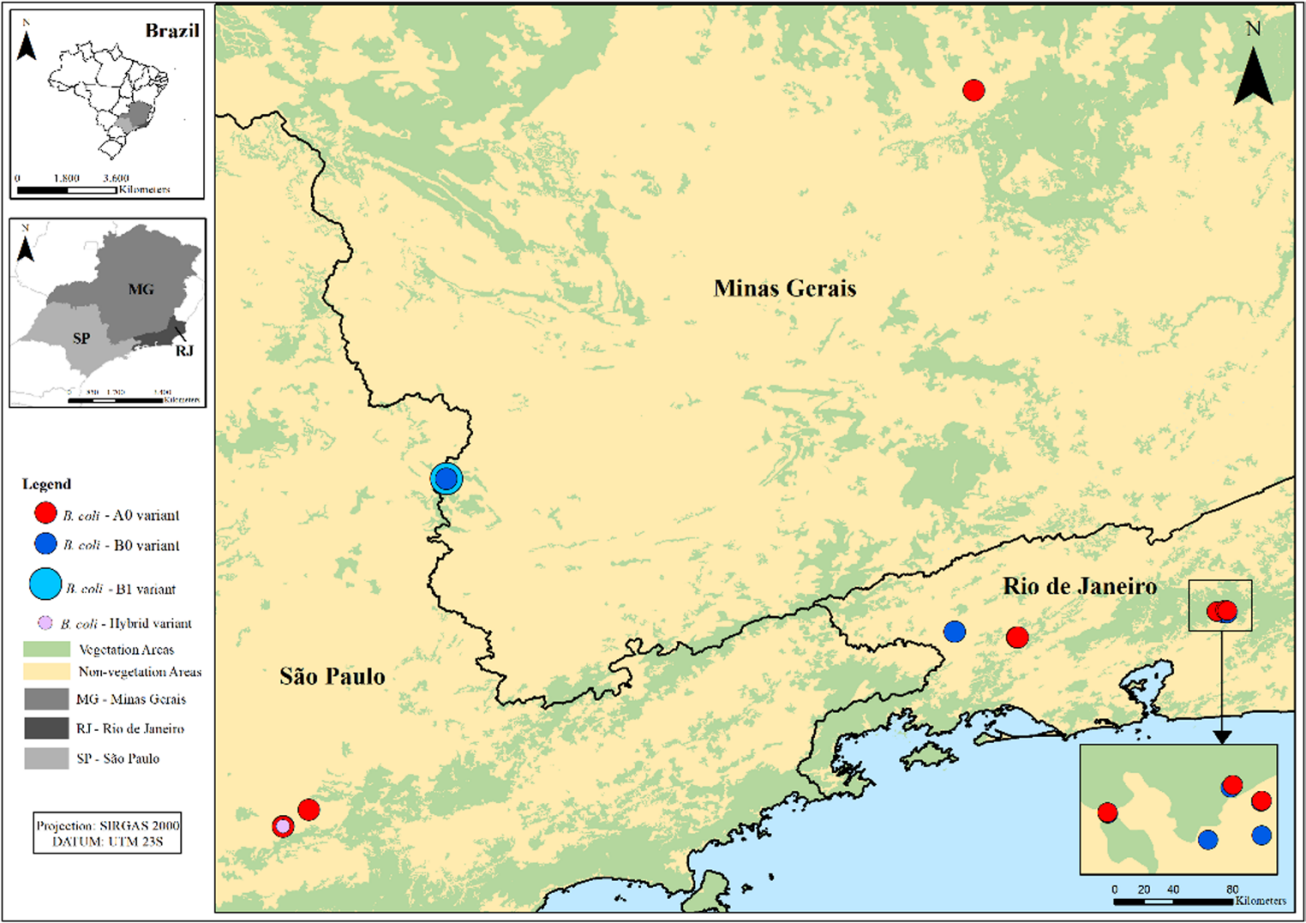
Spatial distribution of *Balantioides coli* genetic variants identified in *Tapirus terrestris* under different management contexts (*ex situ* and *in situ*) in Southeastern Brazil

Fecal samples from *Tapirus terrestris* were collected between March 2023 and June 2025. Samples were obtained from tapirs reintroduced into the Guapiaçu Ecological Reserve (REGUA), located in Cachoeiras de Macacu, Rio de Janeiro, Brazil, as well as from individuals undergoing acclimatization in paddocks prior to release into the wild. Additional samples were collected from tapirs maintained under human care in zoos and wildlife holding facilities located in the states of Rio de Janeiro, São Paulo, and Minas Gerais (Fig. 1).

To preserve institutional confidentiality, all sampling locations were identified using alphabetical codes. Samples were collected from institutions located in Volta Redonda, Rio de Janeiro (A); Piraí, Rio de Janeiro (B); Sorocaba, São Paulo (C); Cotia, São Paulo (D); Itu, São Paulo (E); Poços de Caldas, Minas Gerais (G); and Belo Horizonte, Minas Gerais (H), each representing a single, homogeneous sampling context. In contrast, samples from REGUA (F) were deliberately subdivided into three groups (F1–F3) because they represent distinct biological and environmental conditions: F1 corresponds to animals in an acclimatization paddock (managed, transitional condition), F2 includes necropsy-derived samples (post-mortem condition), and F3 comprises fecal samples collected along trails, with no direct observation or identification of the animals, and therefore attributed to free-ranging individuals (natural, unmanaged condition). This subdivision was required to avoid pooling non-comparable sample types.

The Guapiaçu Ecological Reserve comprises five Private Natural Heritage Reserves, totaling approximately 10,000 hectares dedicated to biodiversity conservation within the Atlantic Forest biome. The tapir reintroduction program at REGUA began in 2018 with the release of three individuals as part of a conservation initiative aimed at restoring the species in the region. Currently, 25 tapirs inhabit the reserve, including individuals born in the wild. Prior to release, animals undergo an acclimatization period in large paddocks located near the Reserve headquarters, characterized by dense Atlantic Forest vegetation and natural water sources. After acclimatization and recovery of natural behaviors, individuals are fitted with radio-telemetry collars and subsequently monitored following release (Galliez et al. 2024).

Tapirs maintained in zoological institutions and wildlife holding facilities were generally housed in large paddocks containing grass, exposed soil, and water sources. The enclosures were open and included covered areas. In most institutions, animals were housed in groups; however, some facilities maintained individuals in separate enclosures. Animals had *ad libitum* access to water and were fed diets consisting of fruits, vegetables, and legumes. In some zoological institutions, tapirs shared enclosures with other species, including cervids, testudines, and birds. Antiparasitic treatments were administered at least every six months, with ivermectin being the most commonly used drug.

### Sampling and fecal sample collection

This study employed a convenience sampling strategy and followed a cross-sectional design. The number of samples analyzed corresponded to those made available by the institutions maintaining the animals, as well as samples obtained from acclimatization paddocks, trail surveys within the reserve, and from a necropsied individual found in the reintroduction area in REGUA.

Fecal samples, except for those obtained during necropsy, were collected directly from the floor of enclosures in zoological institutions and wildlife holding facilities, from acclimatization paddocks, and from the ground along trails within the reserve. In zoos, holding facilities, and acclimatization paddocks, sample collection was previously scheduled with the animal care staff of each institution. Fresh fecal samples were prioritized, particularly well-defined fecal piles. Each sample was placed in a labeled transparent plastic bag corresponding to the respective enclosure. Whenever possible, to allow individual identification, tapirs were separated one day prior to and kept overnight in handling facilities or in individual enclosures.

Trail surveys within the reserve were conducted with the assistance of a local resident familiar with the area and involved in other REGUA projects. Defecation sites commonly used by tapirs had been previously identified and marked with ribbons attached to nearby trees. During these surveys, fecal samples were obtained from two distinct sites. Sampling locations were georeferenced using a Garmin eTrex 20 GPS device, and samples were photographed with identification tags before collection. Subsequently, samples were stored in labeled plastic bags corresponding to tag identification numbers. During the study period, in June 2023, a free-ranging female tapir was found dead within the reserve and was subjected to field necropsy. Five fecal samples collected from this individual were included in the study.

All samples were immediately transported to the Parasitology Laboratory of the Biomedical Institute at the Federal Fluminense University in thermal containers for further processing and analysis. Whenever feasible, preliminary sample processing, including inoculation into culture medium, was initiated at the collection sites.

A total of 45 fecal samples were collected in this study. Of these, 20 samples were obtained from tapirs in the Guapiaçu Ecological Reserve (REGUA). Among these, seven were obtained from individuals undergoing acclimatization in paddocks prior to release (F1), five samples were collected from a carcass found along a trail (F2) and eight were collected from fecal deposits on the forest trails (F3). Additionally, 25 fecal samples were collected from tapirs maintained under human care. These samples were obtained from the following institutions: A (*n* = 1); B (*n* = 2); C (*n* = 7); D (*n* = 5); E (*n* = 2); G (*n* = 4); and H (*n* = 4). The number of fecal samples collected, and the number of tapirs present at each sampling location were presented in Supplementary Table 1.

### Detection of gastrointestinal parasites by microscopy

A portion of each fecal sample was subjected to direct examination to detect trophozoites, with an emphasis on ciliates. The remaining portion of the fecal material was processed using parasitological techniques, including centrifugal sedimentation (Ritchie 1948; modified by Young et al. 1979), centrifugal flotation using a sucrose solution with a density of 1,200 g/mL (Sheather 1923; modified by Huber et al. 2003), and spontaneous sedimentation (Lutz 1919), to identify structures compatible with parasites (helminths or protozoa).

Slides obtained from each technique were examined microscopically, and morphometric evaluation of parasitic forms and photomicrography were performed using a binocular optical microscope (Olympus^®^ BX41). Observations were initially conducted at 100× magnification and, when necessary for confirmation, at 400× magnification. Images were captured using a BEL^®^ EU12CONVS digital camera.

### Isolation and maintenance of ciliates in vitro

All collected fecal samples were inoculated into a xenic Pavlova culture medium (Pavlova 1938) modified according to Jones (1946), supplemented with different serum sources to maximize the recovery and maintenance of ciliates belonging to the phylum Ciliophora. Two types of modified Pavlova media were prepared, differing in serum composition: modified Pavlova medium supplemented with fetal bovine serum (PB) and modified Pavlova medium supplemented with equine serum (PC). Two serum sources were used to improve isolation success, considering the potential variability in the adaptation of ciliates to in vitro conditions. Preparation of complete culture medium, serum concentrations, antibiotic solutions, and supplementation with rice starch suspension, was performed according to previously described protocols (Class et al. 2024). Parasite isolation and maintenance were performed according to the protocol previously described by our research group (Barbosa et al. 2015).

### Morphological analysis of Ciliophora isolates

All isolates maintained in culture for more than one month were deprived of rice starch supplementation for two consecutive days. Isolates that survived this period were subsequently subjected to morphological analysis. For this analysis, a drop of sediment from each culture tube was placed on a microscope slide and covered with a 24 × 32 mm coverslip. For each isolate, the morphology of 30 trophozoites was analyzed.

### DNA extraction and polymerase chain reaction (PCR) for protozoan detection

All isolates that remained viable for more than five days in modified Pavlova medium were subjected to DNA extraction for molecular screening of ciliates belonging to the phylum Ciliophora. In addition, fecal samples from which ciliates were not successfully isolated in vitro were also subjected to molecular analysis using DNA extracted directly from fecal material. The genomic DNA from both cultured isolates and fecal samples was extracted using the QIAamp^®^ DNA Stool Mini Kit (Qiagen, Germany). For cultured isolates, a 200 µL aliquot of the culture sediment was used for DNA extraction. For fecal samples, approximately 200 mg of fecal material was processed. All extraction procedures followed the manufacturer’s instructions, with two modifications in the incubation steps as previously described (Pinheiro et al. 2023). Extracted DNA was stored at − 20 °C until use in polymerase chain reaction (PCR) assays.

The extracted DNA was subjected to PCR amplification using Platinum™ Hot Start PCR Master Mix (Invitrogen^®^, USA) and primers targeting the ITS1–5.8SrDNA–ITS2 region of ciliate protozoa, as previously described (Ponce-Gordo et al. 2008). PCR reactions were performed in a final volume of 25 µL, containing 5 µL of extracted DNA as template. This volume was established based on prior standardization in our laboratory and successfully applied in previous studies (Class et al. 2026), ensuring sufficient template concentration for fecal samples.

In addition to Ciliophora screening, PCR assays targeting members of the phylum Amoebozoa were also performed, as amoeboid forms were unexpectedly observed in some isolates maintained in vitro. For Amoebozoa detection, primers targeting the 18 S rRNA gene were employed. The detection of *Entamoeba histolytica*, *E. dispar*, and *E. moshkovskii* was performed using nested PCR protocols previously described (Paglia and Visca 2004; Lau et al. 2013). Molecular screening for *E. coli*, *E. polecki*, *E. hartmanni*, *Endolimax nana*, and *Iodamoeba* spp. was performed using conventional PCR based on the same primary PCR protocol used for *E. histolytica*, *E. dispar*, and *E. moshkovskii*. However, cycling conditions were adjusted according to previously described protocols (Stensvold et al. 2011, 2018; Chihi et al. 2019). Additionally, molecular detection of *E. suis* was performed using a nested PCR approach following previously described protocols (Clark et al. 2006; Wang et al. 2022).

All amplification reactions were carried out in an Applied Biosystems thermocycler (Life Technologies^®^, USA) following cycling conditions described in the respective references. PCR products were visualized by electrophoresis on 1.5% agarose gels stained with GelRed^®^ (Biotium, USA) and bromophenol blue (LGC^®^, UK). Negative controls consisting of ultrapure water and positive controls consisting of *Balantioides coli* culture maintained in our laboratory and previously confirmed Amoebozoa-positive samples were included in all DNA extraction and PCR assays (Dos Santos et al. 2025; Dib et al. 2025a). PCR products were purified using ExoSAP-IT™ (Invitrogen^®^, USA) and sequenced at the Fiocruz sequencing platform.

### Nucleotide sequence analysis of ciliate protozoa

Initial sequence inspection and editing were performed using ChromasPro v.1.7.5. Subsequently, nucleotide sequences were compared with in the GenBank database using the BLASTn tool to identify homologous sequences corresponding to the same gene fragment. All sequences were saved in FASTA format and aligned with homologous sequences retrieved from GenBank using BioEdit software for both Ciliophora and Amoebozoa groups, in order to determine parasite species.

For the Ciliophora and Amoebozoa group, phylogenetic inference was performed using the Maximum Likelihood (ML) method with bootstrap support calculated from 1,000 replicates. The best-fit evolutionary model was selected based on the Akaike Information Criterion (AIC) using the online platform W-IQ-TREE (http://iqtree.cibiv.univie.ac.at/). The resulting phylogenetic tree was edited and rooted using MEGA X and iTOL.

Reference sequences of *Balantioides coli* and its genetic variants (A0, A1, A2, B0, and B1) from different hosts available in GenBank were included in the analysis. In addition, sequences from other ciliate species were incorporated as outgroups, including morphologically similar protozoa such as *Buxtonella* sp., *Buxtonella sulcata*, and *Balantidium entozoon* (Pomajbíková et al. 2013; Grim et al. 2009). More distantly related ciliates, including *Troglodytella abrassarti* and *Isotricha prostoma*, were also included to root the phylogenetic tree (Wright 1999; Modrý et al. 2009).

Reference sequences of species belonging to the genus *Entamoeba* available in GenBank were included in the phylogenetic analysis, encompassing isolates from different hosts and geographic regions, including *Entamoeba histolytica*, *Entamoeba dispar*, *Entamoeba moshkovskii*, *Entamoeba suis*, *Entamoeba coli*, *Entamoeba polecki*, and *Entamoeba hartmanni* (Sogin et al. 1992 retrieved from Genbank; Novati et al. 1995 retrieved from Genbank; Silberman et al. 1999; Clark et al. 2006; Lai 2024 retrieved from Genbank). Sequences generated were compared with these reference isolates to evaluate their phylogenetic positioning and genetic relatedness. *Giardia duodenalis* was included as the outgroup taxon (Morrison et al. 2007).

### PCR amplification and nucleotide sequence analysis used for host identification

Fecal samples collected along trails within the Guapiaçu Ecological Reserve were also subjected to molecular analysis to confirm the host species associated with the fecal material. DNA previously extracted from these samples, as described above, was used as template in PCR assays targeting a fragment of approximately 710 bp of the mitochondrial cytochrome c oxidase subunit I (COI) gene. Amplification was performed using universal primers described and protocol originally proposed Folmer 1994), which is widely used for molecular identification across multiple mammalian orders. Negative controls consisting of ultrapure water and positive controls consisting of fecal material from *Tapirus terrestris* collected at an ecological refuge located in Itu, São Paulo, were included in all DNA extraction and PCR procedures.

PCR products were purified using ExoSAP-IT™ enzyme (Invitrogen^®^) and sequenced at the CREBIO platform (Jaboticabal, Brazil). The resulting nucleotide sequences were aligned and edited using BioEdit v7.2.5 and subsequently compared with reference sequences of *Tapirus terrestris* deposited in GenBank, accession number GU737554.1 (Cozzuol et al. 2013), as well as with the sequence generated from the positive control included in the present study.

### Data analysis and presentation of results

Parasitological findings were initially summarized in tables, with parasites reported at the lowest possible taxonomic level for each sampling location. The frequency of each parasitic taxon was calculated based on the total number of samples analyzed and the number of positive samples detected at each site. To evaluate potential differences in parasite distribution among sampling locations, comparative statistical analyses were performed using chi-square tests based on the total number of samples and the number of positives. P-values were calculated separately for each parasitic group, adopting a significance level of 5% (*p* < 0.05). All statistical analyses were performed using Epi Info software.

Molecular results were integrated with microscopic findings and host identification to estimate the overall frequency and distribution of the parasitic agents detected in the analyzed samples. Morphometric parameters of ciliates were assessed based on cell length and width, as well as the length, width, shape, and position of the macronucleus. All morphometric measurements are reported in the Results section as minimum and maximum values, mean and standard deviation.

## Results

### Host Identification

Host identification based on the mitochondrial COI gene was performed exclusively for the eight fecal samples collected along trails within the reserve (F3), as these samples were obtained directly from the ground without visual confirmation of the animals. In contrast, for all other samples, host identity was established through direct observation of the defecating individuals. Of the eight F3 samples, five yielded nucleotide sequences of 425 base pairs suitable for analysis. All sequences showed 100% identity with *Tapirus terrestris* based on the positive control and 99.76% identity with the reference sequence deposited in GenBank (accession number GU737554.1). The corresponding results are presented in Supplementary Fig. 1.

### Overall parasitological findings

Microscopic parasitological examination revealed that 31 of the 45 fecal samples (68.9%) were positive for protozoa or helminths. The overall positivity varied among sampling sites, with no parasites detected at site D and maximum frequencies (100%) observed at sites A, B, E, and F3. Among the detected parasites, protozoa belonging to the phylum Ciliophora were the most frequently observed, with cysts or trophozoites identified in 27 samples (60%). These forms were detected at all sampling sites except site D. High frequencies were recorded at sites A, B, C, F1, and F3 (≥ 85%), whereas lower frequencies were observed at sites F2 and H, indicating significant variation among sampling locations (χ², *p* = 0.0173) (Table 1).

In addition to ciliates, other protozoa were detected, including non-sporulated coccidian oocysts with an average size of 21.48 ± 4.24 μm in length and 21.48 ± 4.24 μm in width, as well as cysts of *Entamoeba* spp., measuring approximately 28.8 μm in diameter. These protozoa were observed exclusively in fecal samples from animals maintained under human care in institutions C, G, and H. The occurrence of coccidian oocysts differed significantly among sampling sites (χ², *p* = 0.0014), whereas *Entamoeba* spp. cysts were rare, being detected in only one sample and with no significant spatial variation observed (*p* = 0.3992) (Table 1).

Regarding helminths, nematode forms were primarily identified as thin-shelled eggs observed in fecal samples from tapirs maintained under human care at sites C, E, F1, and H, as well as from reintroduced individuals within the reserve (sites F2 and F3). These eggs measured between 32 and 110 μm in length (mean 55.39 ± 24.98 μm) and between 19.2 and 64 μm in width (mean 31.56 ± 10.63 μm) and were morphologically compatible with strongyle-type eggs. These structures were detected in 14 samples (31.1%) and were distributed across sampling sites without statistically significant differences (χ², *p* = 0.2204). Nematode larvae were detected less frequently (6 samples; 13.3%) and were recorded only in fecal samples from site E and from free-ranging reintroduced tapirs, mainly at site F3, showing a significant difference among sampling locations (χ², *p* = 0.0223). Additionally, eggs belonging to the family Spiruridae and *Trichuris* sp. were identified, both detected exclusively at site G, each with a frequency of two samples (4.4%). Their spatial distribution was statistically significant (χ², *p* = 0.0181 for both taxa) (Table 1).

**Table 1:** Frequency and spatial distribution of gastrointestinal parasites detected by microscopy in fecal samples from *Tapirus terrestris* under human care and reintroduced across sampling sites in southeastern Brazil

Parasites	A (*n* = 1)	B (*n* = 2)	C (*n* = 7)	D (*n* = 5)	E (*n* = 2)	F1 (*n* = 7)	F2 (*n* = 5)	F3 (*n* = 8)	G (*n* = 4)	H (*n* = 4)	Positive samples	*p* value
Ciliophora Group	1(100%)	2(100%)	6(85.7%)	0	1(50%)	5(85.7%)	1(20%)	7(87.5%)	2(50%)	1(25%)	27(60%)	0.0173*
Coccidian oocyst	0	0	6(85.7%)	0	0	0	0	0	2(50%)	1(25%)	9(20%)	0.0014*
Thin-shelled nematode egg	0	0	6(85.7%)	0	1(50%)	2(28.6%)	1(20%)	2(25%)	2(50%)	0	14(31.1%)	0.2204
Nematode larvae	0	0	0	0	1(50%)	0	0	5(62.5%)	0	0	6(13.3%)	0.0223*
Spiruridae egg	0	0	0	0	0	0	0	0	2(50%)	0	2(4.4%)	0.0181*
***Trichuris*** **spp. egg**	0	0	0	0	0	0	0	0	2(50%)	0	2(4.4%)	0.0181*
***Entamoeba*** **spp. cyst**	0	0	0	0	0	0	0	0	1(25%)	0	1(2.2%)	0.3992
Total positivity	1(100%)	2(100%)	6(85.7%)	0	2(100%)	6(85.7%)	2(40%)	8(100%)	2(50%)	2(50%)	31(68.9%)	

A: Volta Redonda, Rio de Janeiro (RJ); B: Piraí, RJ; C: Sorocaba, São Paulo (SP); D: Cotia, SP; E: Itu, SP; F1: Ecological Reserve (RJ), acclimatization phase; F2: Ecological Reserve (RJ), necropsy samples; F3: Ecological Reserve (RJ), trail-collected samples; G: Poços de Caldas, Minas Gerais (MG); H: Belo Horizonte, MG. *Statistically significant (*p* < 0.05)

A total of 18 samples showed successful isolation of parasites belonging to the phylum Ciliophora. Of these, 11 were isolated exclusively in PB medium, five only in PC medium, and two in both media (samples 29 and 31), totaling 20 isolates (Table 2). All isolates yielded amplified products in the polymerase chain reaction (PCR) targeting Ciliophora DNA. In addition, six samples in which DNA was extracted directly from fecal material also produced positive PCR amplification for this eukaryotic group. Agarose gel electrophoresis profiles of selected samples positive for Ciliophora are shown in Supplementary Fig. 2.

**Table 2:** Sampling sites, in vitro isolation, and molecular characterization of Ciliophora and *Entamoeba* spp. detected in fecal samples from *Tapirus terrestris* under human care and reintroduced in southeastern Brazil

Sample	Site	State (Brazil)	Animal condition	Direct examination	*In vitro* culture	PCR	Sequencing	Genetic Variant	Genbank
1	C	São Paulo	*Ex situ*	Cysts	Not isolated	Feces/PCR-	NA	NA	NA
2	C	São Paulo	*Ex situ*	Trophozoites and cysts	Isolated in PB	Culture/PCR+	*Balantioides coli*	A0	PX578977
3	C	São Paulo	*Ex situ*	Trophozoites and cysts	Isolated in PB	Culture/PCR+	*Balantioides coli*	A0	PX578978
5	C	São Paulo	*Ex situ*	Trophozoites	Not isolated	Feces /PCR+	*Balantioides coli*	A and B	NA
6	C	São Paulo	*Ex situ*	Trophozoites and cysts	Isolated in PC	Culture/PCR+	*Balantioides coli*	A0	PX578979
7	C	São Paulo	*Ex situ*	Trophozoites	Not isolated	Feces /PCR-	NA	NA	NA
8	F1	Rio de Janeiro	*Ex situ*	Cysts	Not isolated	Feces /PCR-	NA	NA	NA
9	F1	Rio de Janeiro	*Ex situ*	Cysts	Not isolated	Feces /PCR-	NA	NA	NA
10	F1	Rio de Janeiro	*Ex situ*	Cysts	Isolated in PB	Culture/PCR+	*Balantioides coli*	A0	PX578980
							*Entamoeba coli*		NA
							*Entamoeba hartmanni*		PX458010
12	F1	Rio de Janeiro	*Ex situ*	Cysts	Isolated in PB	Culture/PCR+	*Balantioides coli*	A0	PX578981
							*Entamoeba hartmanni*		PX458011
13	F1	Rio de Janeiro	*Ex situ*	Cysts	Isolated in PC	Culture/PCR+	*Balantioides coli*	A0	PX578982
							*Entamoeba coli*		NA
							*Entamoeba hartmanni*		PX458012
18	F2	Rio de Janeiro	*In situ*	Cysts	Not isolated	Feces /PCR+	*Balantioides coli*	B0	PX578983
19	A	Rio de Janeiro	*Ex situ*	Trophozoites	Isolated in PB	Culture/PCR+	*Balantioides coli*	B0	PX578984
							*Entamoeba coli*		NA
20	F1	Rio de Janeiro	*Ex situ*	Trophozoites	Isolated in PC	Culture/PCR+	*Balantioides coli*	B0	PX578985
							*Entamoeba coli*		NA
							*Entamoeba suis*		PX458013
21	E	São Paulo	*Ex situ*	Cysts	Isolated in PB	Culture/PCR+	*Balantioides coli*	A0	PX578986
28	B	Rio de Janeiro	*Ex situ*	Cysts	Isolated in PB	Culture/PCR+	*Balantioides coli*	A0	PX578987
29	B	Rio de Janeiro	*Ex situ*	Cysts	Isolated in PC	Culture/PCR+	*Balantioides coli*	A0	PX578988
29	B	Rio de Janeiro	*Ex situ*	Cysts	Isolated in PB	Culture/PCR+	*Balantioides coli*	A0	PX578988
30	F3	Rio de Janeiro	*In situ*	Cysts	Not isolated	Feces/PCR+	*Balantioides coli*	B0	PX578989
31	F3	Rio de Janeiro	*In situ*	Cysts	Isolated in PB	Culture/PCR+	*Balantioides coli*	A0	PX578990
31	F3	Rio de Janeiro	*In situ*	Cysts	Isolated in PC	Culture/PCR+	*Balantioides coli*	A0	PX578990
32	F3	Rio de Janeiro	*In situ*	Cysts	Not isolated	Feces/PCR+	*Balantioides coli*	B0	PX578991
33	F3	Rio de Janeiro	*In situ*	Cysts	Not isolated	Feces/PCR+	*Balantioides coli*	B0	PX578992
34	F3	Rio de Janeiro	*In situ*	Trophozoites and cysts	Isolated in PC	Culture/PCR+	*Balantioides coli*	B0	PX578993
35	F3	Rio de Janeiro	*In situ*	Cysts	Isolated in PC	Culture/PCR+	*Balantioides coli*	B0	PX578994
36	F3	Rio de Janeiro	*In situ*	Cysts	Isolated in PB	Culture/PCR+	*Balantioides coli*	A0	PX578995
39	G	Minas Gerais	*Ex situ*	Negative	Isolated in PB	Culture/PCR+	*Balantioides coli*	B1	PX578996
							*Entamoeba hartmanni*		PX458014
40	G	Minas Gerais	*Ex situ*	Trophozoites	Isolated in PB	Culture/PCR+	*Balantioides coli*	B1	PX578997
							*Entamoeba coli*		NA
41	G	Minas Gerais	*Ex situ*	Trophozoites	Isolated in PB	Culture/PCR+	*Balantioides coli*	B0	PX578998
							*Entamoeba hartmanni*		PX458015
41	G	Minas Gerais	*Ex situ*	Negative	Isolated in PC	Culture/PCR+	*Entamoeba hartmanni*	NA	PX458016
42	H	Minas Gerais	*Ex situ*	Cysts	Not isolated	Feces/PCR+	*Balantioides coli*	A0	PX578999

*PB* Pavlova biphasic medium, *PC* Pavlova complete medium, *PCR+* positive PCR result, *PCR−* negative PCR result, *NA* not available, *Ex situ* animals maintained under human care, *In situ* free-ranging or reintroduced animals. Genetic variants refer to ITS1–5.8 S–ITS2 sequence profiles of *Balantioides coli*

### *Balantioides coli*: molecular detection and variant distribution

Molecular analysis confirmed the presence of *Balantioides coli* in 24 out of 45 samples analyzed (53.3%), including isolates obtained both directly from fecal material and from in vitro cultures. All sequences clustered within the *B. coli* clade, showing identity values ranging from 98.08% to 100% when compared with reference sequences available in GenBank. Phylogenetic analysis revealed the occurrence of three genetic variants, A0 (50%), B0 (37.5%), and B1 (8.3%), with a clear predominance of A0. Variant A0 was widely distributed across sampling sites and occurred in both *ex situ* and *in situ* populations. Additionally, one sample exhibited a mixed pattern, suggesting the simultaneous presence of variants A and B. All sequences generated in this study were deposited in GenBank under accession numbers PX578977–PX578999.

**Fig. 2:**
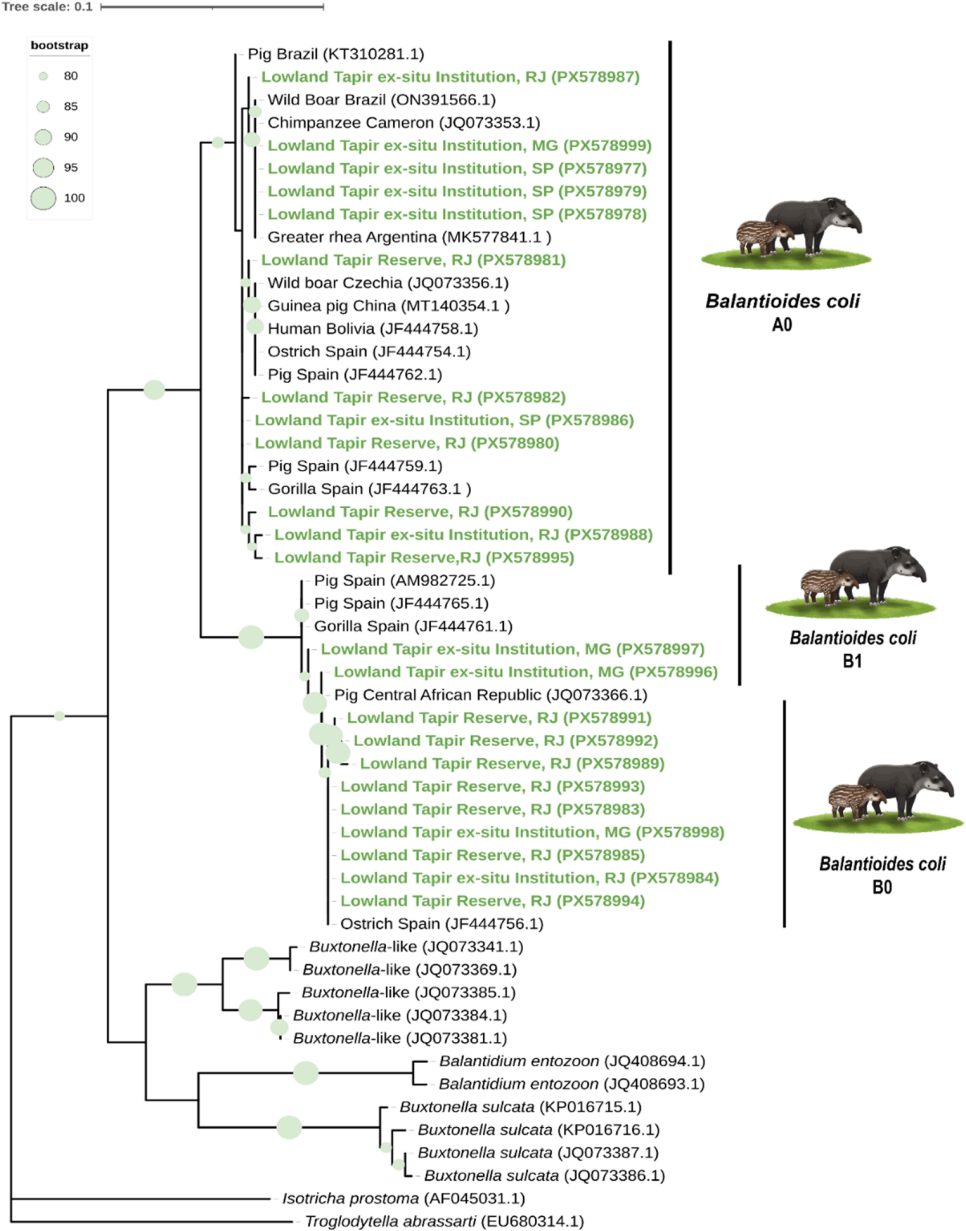
Maximum likelihood phylogenetic tree based on ITS sequences illustrating the genetic relationships of *Balantioides coli* isolates obtained from *Tapirus terrestris* in southeastern Brazil and reference sequences retrieved from GenBank. The isolates generated in this study are highlighted in green. The sequences clustered within the previously described genetic variants A0, B0, and B1. Bootstrap support values (1000 replicates) are indicated at the nodes. *Buxtonella sulcata*, *Balantidium entozoon*, *Isotricha prostoma*, and *Troglodytella abrassarti* were included as outgroup taxa

In the phylogenetic tree (Fig. 2), the isolates generated in this study clustered into two main clades corresponding to the previously described genetic variants A0 and B0 of *B. coli*. Sequences classified as A0 grouped together with reference sequences obtained from different hosts and geographic regions, forming a well-supported cluster with low intra-clade divergence. Similarly, isolates belonging to variants B0 and B1 were positioned within the B clade, clustering closely with sequences from other hosts and geographic regions.

Spatial analysis revealed a broad distribution of *B. coli* genetic variants associated with *T. terrestris* across the states of Rio de Janeiro, São Paulo, and Minas Gerais (Fig. 1), including records from animals maintained under human care as well as from individuals reintroduced or already established in free-ranging conditions within the Guapiaçu Ecological Reserve. The genetic variants A0 and B0 were detected under both management conditions, whereas variant A0 showed the widest geographic distribution, being recorded in all three evaluated states. In contrast, variant B0 exhibited a more restricted distribution and was detected only in samples from Rio de Janeiro and Minas Gerais. Sequences belonging to variant B1 were identified exclusively in isolates obtained from tapirs maintained under human care in Minas Gerais.

### Morphological analysis of cultured protozoa

**Fig. 3:**
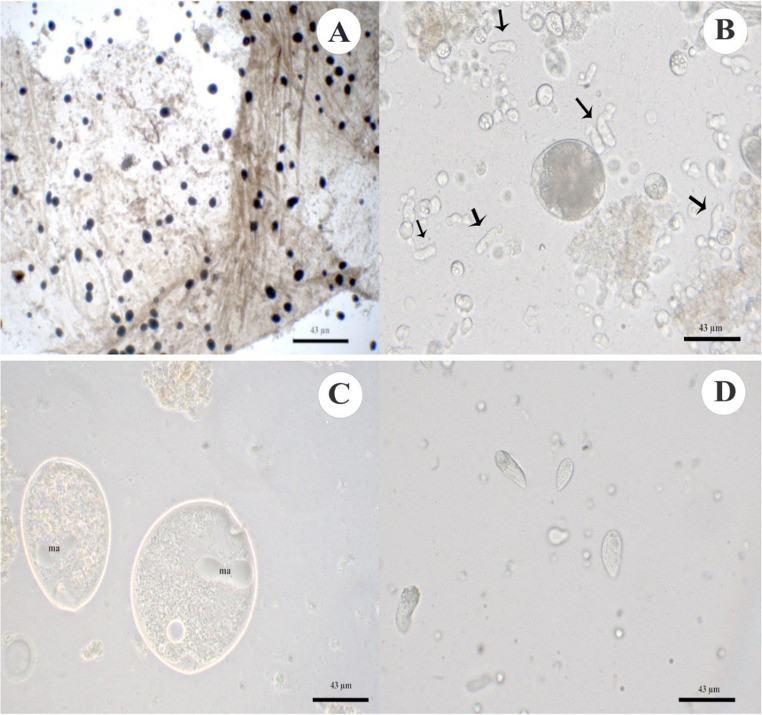
Trophozoites of protozoan parasites observed in in vitro cultures using modified Pavlova medium isolated from fecal samples of *Tapirus terrestris*. In (A), ciliated trophozoites of *Balantioides coli* isolated from samples collected in Sorocaba, São Paulo, are shown at 40× magnification. In (B), a ciliated trophozoite of *B. coli* is visible in the center of the image, while arrowheads indicate forms compatible with the phylum Amoebozoa isolated from fecal samples collected in Volta Redonda, Rio de Janeiro (400× magnification). In (C) and (D), ciliated trophozoites of *B. coli* isolated from fecal samples collected in Sorocaba, São Paulo, and REGUA, Rio de Janeiro, respectively, are shown at 400× magnification. In (C), the macronucleus (*ma)* is highlighted

A total of five isolates molecularly characterized as *B. coli* were successfully maintained in long-term culture and remained viable after rice starch deprivation. The trophozoites of these isolates ranged from 25.6 to 176 μm in length and from 12.8 to 140.8 μm in width. In general, trophozoites from samples 12 and 20, obtained from *T. terrestris* maintained in the acclimatization paddock at the Reserve, showed the smallest measurements compared with the other isolates generated from fecal samples collected within the Reserve and at other sampling sites. Most macronuclei were rod-shaped. Their length ranged from 28.8 to 57.6 μm and their width from 9.6 to 19.2 μm (Fig. 3A–D). In addition to *B. coli*, amoeboid trophozoites were also isolated from four fecal samples, three collected in the Reserve and one obtained from Volta Redonda, Rio de Janeiro (Fig. 3B) Supplementary Table 2.

### Molecular characterization of Amoebozoa

Although this study primarily aimed to isolate members of the phylum Ciliophora, amoeboid forms were also observed during the in vitro cultivation procedures. In total, eight samples yielded isolates identified as belonging to the phylum Amoebozoa. These isolates were subjected to different PCR assays targeting species-specific genetic markers of *Entamoeba*, *Endolimax nana*, and *Iodamoeba* spp. All isolates were identified as belonging to *Entamoeba* spp., PCR amplification revealed *E. coli*, *E. hartmanni*, and *E. suis* DNA in five, six, and one isolates, respectively. Agarose gel electrophoresis profiles of selected samples positive for *Entamoeba hartmanni* are shown in Supplementary Fig. 3. This identification was confirmed by Sanger sequencing for *E. hartmanni* and *E. suis*. However, confirmation of *E. coli* was not possible because the generated sequences did not yield interpretable electropherograms.

**Fig. 4:**
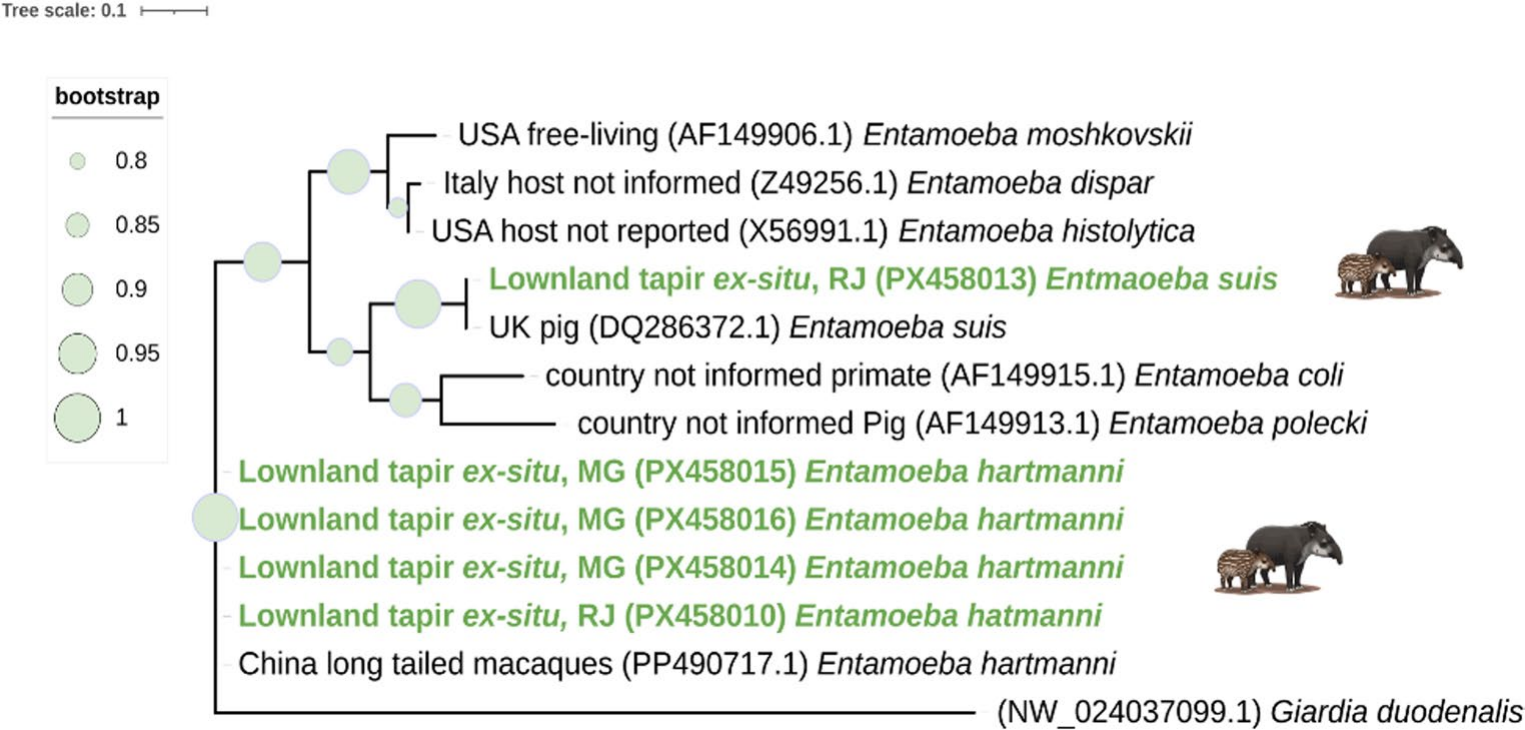
Maximum likelihood phylogenetic tree based on 18 S rRNA gene sequences illustrating the genetic relationships of *Entamoeba* spp. isolates obtained from *ex situ* *Tapirus terrestris* in southeastern Brazil and reference sequences retrieved from GenBank. The isolates generated in this study are highlighted in green. The sequences clustered with reference sequences of *Entamoeba suis* and *Entamoeba hartmanni* previously described from pigs and non-human primates. Bootstrap support values are indicated at the nodes. *Giardia duodenalis* was included as the outgroup taxon

Sequence identity for *E. hartmanni* ranged from 97.6% to 100% when compared with reference sequences available in GenBank (PP490717, MW026789, PP490718, FR686371, MG925066, PP064053, and PP769382), whereas *E. suis* sequences showed identities ranging from 99.6% to 100% relative to reference sequences (DQ286372, PP064056, and PP735759). The sequences of *E. hartmanni* were deposited in GenBank under accession numbers PX458010–PX458012 and PX458014–PX458016, while the *E. suis* sequence was deposited under accession number PX458013. Phylogenetic analysis based on partial 18 S rRNA gene sequences corroborated the molecular identification, with the isolates generated in the present study clustering together with reference sequences of *E. hartmanni* and *E. suis* available in GenBank. *Entamoeba hartmanni* isolates grouped within a clade containing sequences previously described from humans and non-human primates, whereas the *E. suis* isolate clustered with sequences obtained mainly from pig. (Fig. 4).

## Discussion

Overall, the frequency of gastrointestinal parasites detected in *T. terrestris* fecal samples reached 68.9% when combining individuals under human care and free-ranging animals from the Guapiaçu Ecological Reserve. This rate falls within the range previously reported for tapirs, although substantial variation was observed across different contexts. When considering only *ex situ* animals, the positivity observed in the present study (65.6%) was higher than that reported in Parque Dois Irmãos, Brazil (0%), zoological institutions in Madrid, Spain (8.3%), and the Rio de Janeiro Zoo (50%) (Barbosa et al. 2020; Esteban-Sánchez et al. 2024; Freitas et al. 2001), but lower than the 100% frequencies described in institutions in Bolivia and in the Brazilian states of Paraíba and Bahia (Batista et al. 2021; Saavedra et al. 2009; Silva et al. 2022). Among free-ranging tapirs, the parasite frequency observed here (76.9%) was higher than that reported for *Tapirus bairdii* in Mexico (approximately 68.4%) and for *T. terrestris* in the Brazilian Cerrado (50%) (Cruz-Aldán et al. 2006; Fernandes-Santos et al. 2020), but lower than the values documented for *T. bairdii* in forest ecosystems in Mexico (84%) and mountainous regions of Costa Rica (98.6%) (Alvarado-Palacios 2018; Romero-Castañon et al. 2008). These differences across studies likely reflect variations in sample size, which are often constrained in both captive and wild populations, particularly when dealing with threatened and elusive species (Batista et al. 2021; Saavedra et al. 2009; Silva et al. 2022; Dib 2019).

This situation is particularly relevant for *T. terrestris*, which is currently classified as vulnerable to extinction (Medici et al. 2012). Furthermore, differences in parasite occurrence may also reflect methodological variation among studies, including the diagnostic techniques applied, as well as the susceptibility of this host species to infection by diverse parasitic taxa, encompassing both helminths and protozoan organisms.

It is noteworthy that parasites were detected in fecal samples from tapirs present in all surveyed institutions, except for Institution D, where no parasitic forms were observed using the parasitological microscopic techniques applied. Although all institutions reported having veterinary medical support, providing antiparasitic treatments, or performing routine parasitological diagnostics, differences in parasite occurrence among institutions were evident. This discrepancy may be directly related to the enclosure characteristics at Institution D, where the tapirs spend part of their daily activity period. The enclosure consists of a closed artificial environment designed to mimic a small forest and includes a cascading river supplied with treated and regularly monitored water, in which the animals move freely. Such environmental management conditions may contribute to reduced exposure to infective parasitic stages. However, the differences in parasite occurrence among the institutions maintaining tapirs under *ex situ* conditions should be interpreted with caution, as this study relied on a single-point collection of biological samples. Parasitic forms may be shed intermittently in fecal material, potentially influencing detection rates.

Overall, protozoa were detected more frequently than helminths. This pattern was expected, as the antiparasitic drugs commonly administered to the animals, particularly ivermectin, frequently reported by the caretakers, are primarily effective against nematode infections but generally have limited activity against protozoan parasites. Among the detected parasites, members of the phylum Ciliophora, including both cysts and trophozoites, represented the most frequently observed forms in the parasitological microscopy examinations.

These ciliates were identified both in tapirs reintroduced into the Reserve and in individuals maintained *ex situ* in different zoological institutions located in Rio de Janeiro, São Paulo and Minas Gerais states. Consistent with these findings, developmental stages of this ciliate have previously been reported in parasitological surveys involving *T. terrestris* maintained under *ex situ* conditions in different breeding facilities in Brazil and Spain. Similar records have also been described in samples from *Tapirus indicus* (Malayan tapir) in Malaysia, as well as in *T. bairdii* under *in situ* conditions in Mexico and Costa Rica, and in *T. terrestris* individuals found as roadkill in Brazil (Alvarado-Palacios 2018; Barbosa et al. 2020; Batista et al. 2021; Cruz-Aldán et al. 2006; Esteban-sánchez et al. 2024; Mangini et al. 2002; Mustapa et al. 2014; Navas-Suárez et al. 2019).

Despite the predominance of forms compatible with protozoa belonging to the phylum Ciliophora in fecal samples obtained from different institutions, significant differences in positivity were observed among the evaluated locations. This pattern indicates that the occurrence of these ciliates is not homogeneous, reflecting ecological and sanitary heterogeneity among environments. High frequencies recorded at locations A, B, C, F1, and F3 (≥ 85%) suggest environmental conditions favorable for the maintenance of transmission, whereas the lower prevalences observed at F2 and H may be associated with improved sanitary management practices, lower host density, or microenvironmental differences capable of reducing the viability of infective stages.

Molecular characterization of these ciliates, derived from isolates obtained both from in vitro cultures and directly from fecal samples, confirmed the presence of *Balantioides coli* in 24 out of 45 analyzed samples, corresponding to an infection rate of approximately 53.3%. The obtained sequences exhibited high identity values when compared with isolates previously described from a wide range of hosts and geographic regions, including domestic pigs, feral pigs, non-human primates, ostriches, and humans from South America, Europe, and Africa (Barbosa et al. 2017; Ponce-Gordo et al. 2008, 2011; Pomajbíková et al. 2013; García-Rodríguez et al. 2020; Pinheiro et al. 2023), reinforcing the generalist nature of the parasite and its capacity to circulate among multiple host species. The relatively high infection rate observed in *T. terrestris* highlights the epidemiological relevance of this ciliate in this host, particularly when compared to previous studies based exclusively on morphological identification, which may underestimate its occurrence due to diagnostic limitations (Alvarado-Palacios 2018; Barbosa et al. 2020; Batista et al. 2021; Cruz-Aldán et al. 2006; Esteban-Sánchez et al. 2024; Mangini et al. 2002; Mustapa et al. 2014; Navas-Suárez et al. 2019). Notably, this infection pattern appears to be strongly influenced by the distribution of specific genetic variants.

The analysis of genetic variants revealed the presence of A0, B0, and B1 types, with a clear predominance of A0, which was widely distributed across both *ex situ* and *in situ* populations. This variant was detected in tapirs maintained under human care and in free-ranging individuals from Rio de Janeiro and São Paulo, indicating a broad ecological distribution. This pattern is consistent with previous studies conducted in Brazil, in which A0 has also been the most frequently identified variant in pigs and non-human primates (Barbosa et al. 2017; Dib et al. 2025a), and has been reported in multiple hosts worldwide. From a One Health perspective, the predominance of A0 is particularly relevant, as this lineage has already been reported in human samples in South America, suggesting potential zoonotic relevance (Ponce-Gordo et al. 2011; Silva et al. 2021; Class et al. 2026). In contrast, the detection of the B0 variant, although less frequent, has important epidemiological implications, as this genotype is strongly associated with domestic and feral pigs, recognized as the main reservoirs of *B. coli*, and has been widely reported in pig production systems and their handlers (Pinheiro et al. 2023; Dib et al. 2025a, b; Class et al. 2024, 2026). The presence of B0 in tapirs suggests environmental exposure to contaminated areas, particularly in regions with a historical presence of small-scale pig farming, such as the Guapiaçu Ecological Reserve, supporting the hypothesis of cross-species transmission at the wildlife–livestock interface. Variant B1 was detected at low frequency and exclusively in animals maintained under human care, which may indicate a more restricted ecological distribution. Importantly, this study provides the first molecular confirmation of *B. coli* infection in *T. terrestris*, establishing this species as a previously unrecognized host and expanding the known host range of this zoonotic parasite.

Successful in vitro isolation of *B. coli*, with motile trophozoites observed up to the fifth day of incubation in modified Pavlova medium, directly contributed to improving the efficiency of the molecular analyses. Amplification from DNA extracted from cultured isolates reduces the interference of inhibitors commonly present in fecal material and increases the availability of target DNA, resulting in electropherograms with well-defined peaks and high-quality sequence reads. This condition is essential for reliable taxonomic identification, particularly in host species that remain poorly investigated for this parasite (Barbosa et al. 2017; Class et al. 2024).

During the culture period, a broad morphological variation among ciliated trophozoites was observed, including isolates of reduced dimensions that could only be adequately visualized under 400× magnification. This finding is consistent with classical descriptions of intraspecific morphological variation (Zaman 1978) and may reflect adaptations associated with the herbivorous diet and the particularities of the intestinal microbiome of *T. terrestris*. Such observations suggest possible phenotypic plasticity of the parasite in distinct digestive niches.

*Balantioides coli* was detected both in animals maintained under human care and in individuals reintroduced into natural environments, revealing a high frequency of gastrointestinal parasites and the concurrent occurrence of multiple parasitic taxa. The molecular identification of *B. coli* genetic variants previously reported in humans and other hosts, together with the successful in vitro isolation of the parasite, reinforces the potential role of the tapir as a host contributing to the ecological maintenance of this ciliate and highlights the epidemiological relevance of this threatened species.

In addition to *B. coli*, diverse of gastrointestinal parasites were detected in *T. terrestris*, including coccidian oocysts morphologically compatible with *Eimeria* spp., nematode forms—predominantly strongylid-type eggs and larvae, as well as *Trichuris* spp. and members of the family Spiruridae—and amoebae of the genus *Entamoeba*. Collectively, these findings reinforce the notion that *T. terrestris*, similar to other large herbivorous mammals, hosts a complex and multi-taxa parasitic community shaped by environmental exposure and ecological interactions. The low number of coccidian oocysts observed in fecal samples suggests predominantly subclinical infections, which is consistent with previous reports indicating that many *Eimeria* spp. infections in wild and captive hosts may occur without overt clinical signs, particularly under stable host–parasite equilibrium conditions. In contrast, the detection of strongylid-type eggs and larvae reflects transmission dynamics closely linked to environmental contamination, as these nematodes typically present direct life cycles with infective stages developing in soil. This pattern is consistent with the foraging behavior of *T. terrestris*, which increases the likelihood of ingestion of infective stages during grazing and contact with contaminated substrates.

The occurrence of *Trichuris* spp. and Spiruridae, although at lower frequencies, provides additional insight into the diversity of helminth infections in this host and may be influenced by specific ecological and host-related factors, including age, immune status, and enclosure characteristics. In particular, the detection of these taxa in animals maintained under conditions with exposed soil and vegetation may favor the persistence and transmission of infective stages, especially for geohelminths such as *Trichuris* and for parasites with indirect life cycles, such as Spiruridae, which depend on arthropod intermediate hosts.

Additionally, the identification of *Entamoeba* spp., initially observed during in vitro culture and subsequently confirmed by molecular analysis, highlights an important methodological aspect of the present study. It is important to note that the detection of amoebae was not a primary objective of this investigation, and therefore molecular screening was not systematically performed on all original fecal samples prior to cultivation. Instead, the observation of amoeboid trophozoites emerged during culture procedures targeting ciliates, which motivated subsequent molecular identification. The use of xenic culture systems may enhance the detection of protists that are not readily identified by conventional parasitological techniques, revealing components of the intestinal protozoan community that might otherwise remain undetected. However, it should also be considered that not all amoebae present in the original fecal samples are expected to grow and proliferate under modified Pavlova culture conditions, as different taxa exhibit variable requirements and adaptation to in vitro environments. The detection of species such as *Entamoeba hartmanni*, *E. coli*, and *E. suis* further suggests potential interspecific transmission and reinforces the relevance of these findings from a One Health perspective, particularly considering that some of these taxa are recognized as zoonotic or potentially pathogenic in other hosts.

Although these parasites were not the primary focus of this study, their integrated analysis contributes to a more comprehensive understanding of parasite diversity, transmission routes, and host–parasite interactions in *T. terrestris*, emphasizing the importance of considering the broader parasitic community when investigating zoonotic protozoa in wildlife.

From a One Health perspective, the results suggest the existence of an epidemiological interface involving wildlife, anthropogenic environments, and recognized reservoirs, particularly domestic pigs and invasive feral pigs, which may act as sources of environmental contamination in areas where tapirs are being reintroduced. Accordingly, these findings underscore the importance of integrating conservation initiatives with wildlife health monitoring and environmental epidemiological surveillance, particularly in ecosystems experiencing increasing anthropogenic pressure and where interspecific transmission risks may be amplified.

## Supplementary Information

Below is the link to the electronic supplementary material.

Supplementary Figure 1. Uncropped agarose gel electrophoresis images of PCR products targeting the mitochondrial COI gene for host identification.

High Resolution (TIF 635 KB)

Supplementary Figure 2. Uncropped agarose gel electrophoresis images of PCR products targeting the ITS1–5.8S–ITS2 region of Ciliophora for molecular detection

High Resolution (TIF 1.09 MB)

Supplementary Figure 3. Uncropped agarose gel electrophoresis images of PCR products targeting the 18S rRNA gene for molecular detection of *Entamoeba hartmanni*.

High Resolution (TIF 999 KB)

Supplementary Table 1

Supplementary Table 2

## Data Availability

No datasets were generated or analysed during the current study.
