# Supplementary material for: First molecular detection and phylogenetic characterization of Balantioides coli in lowland tapir (Tapirus terrestris)
Source: Parasitol Res. 2026 Jun 11;125(1):100. doi: 10.1007/s00436-026-08707-0 (PMC13478035; doi:10.1007/s00436-026-08707-0)
Supplement: Supplementary file 1 [file 436_2026_8707_MOESM4_ESM.docx]

Table 1. Distribution of *Tapirus terrestris* individuals and fecal samples collected at each sampling site in southeastern Brazil.

| **Sampling sites** | **M** | **F** | **Total individuals** | **Fecal samples** |
| --- | --- | --- | --- | --- |
| A | 2 | 0 | 2 | 1 |
| B | 2 | 0 | 2 | 2 |
| C | 1 | 6 | 7 | 7 |
| D | 2 | 3 | 5 | 5 |
| E | 1 | 1 | 2 | 2 |
| F1 | 1 | 2 | 3 | 7 |
| F2 | 0 | 1 | 1 | 5 |
| F3 | NA | NA | NA | 8 |
| G | 1 | 1 | 2 | 4 |
| H | 4 | 0 | 4 | 4 |

Sampling sites and characteristics of the analyzed samples. Sampling sites: A – Volta Redonda, Rio de Janeiro; B – Piraí, Rio de Janeiro; C – Sorocaba, São Paulo; D – Cotia, São Paulo; E – Itu, São Paulo; F1 – REGUA acclimatization paddock (managed animals); F2 – REGUA necropsy samples (post-mortem); F3 – REGUA trail samples, consisting exclusively of fecal samples collected directly from the ground along trails, attributed to free-ranging individuals, without any visual observation or identification of the animals; G – Poços de Caldas, Minas Gerais; H – Belo Horizonte, Minas Gerais. For F3, *n* refers to the number of fecal samples collected, and neither sex nor the number of individual animals could be determined. Abbreviations: M, males; F, females; NA, not applicable.
