# Supplementary material for: First molecular detection and phylogenetic characterization of Balantioides coli in lowland tapir (Tapirus terrestris)
Source: Parasitol Res. 2026 Jun 11;125(1):100. doi: 10.1007/s00436-026-08707-0 (PMC13478035; doi:10.1007/s00436-026-08707-0)
Supplement: Supplementary file 2 [file 436_2026_8707_MOESM5_ESM.docx]

Table 2. Morphological characteristics obtained from the morphometric analysis of *Balantioides coli* isolates maintained in vitro and tolerant to rice starch starvation.

| **Isolate number** | **Collection site** | **Body – Length** | **Body – Width** | **Nucleus – Shape** | **Nucleus – Length** | **Nucleus – Width** |
| --- | --- | --- | --- | --- | --- | --- |
| 28 | B | 60.8 – 172.8 | 48 - 128 | NO: 30 | NO | NO |
|  |  | (102 ± 16.4) | (70.1 ± 11.3) |  |  |  |
| 3 | C | 96 - 176 | 64 - 140.8 | Rod-shaped: 15 | 28.8 - 57.6 (44.5 ± 8.8) | 9.6 - 19.2 (14.2 ± 3.2) |
|  |  | (132.5 ± 17.8) | (95.8 ± 14.8) | Reniform: 6 |  |  |
|  |  |  |  | NO: 9 |  |  |
| 12 | F1 | 25.6 – 54.4 | 12.8 - 25.6 | NO: 30 | NO | NO |
|  |  | (36.3 ± 5.2) | (18.5 ± 3.3) |  |  |  |
| 13 | F1 | 70.4 – 166.4 | 44.8 - 140.8 | Rod-shaped: 11 | 32 - 54.4 (41.4 ± 6.9) | 9.6 - 19.2 (14.5 ± 2.5) |
|  |  | (111.6 ± 21.1) | (79.6 ± 21.6) | Reniform: 6 |  |  |
|  |  |  |  | NO: 13 |  |  |
| 20 | F1 | 38.4 – 70.4 | 19.2 - 38.4 | NO: 30 | NO | NO |
|  |  | (51.6 ± 8) | (30.5 ± 3.9) |  |  |  |

Sampling sites: B – Piraí, Rio de Janeiro; C – Sorocaba, São Paulo; F1 – REGUA, Rio de Janeiro (acclimatization paddock). NO: Not observed.
